# Termite dispersal is influenced by their diet

**DOI:** 10.1098/rspb.2022.0246

**Published:** 2022-05-25

**Authors:** Simon Hellemans, Jan Šobotník, Gilles Lepoint, Martin Mihaljevič, Yves Roisin, Thomas Bourguignon

**Affiliations:** ^1^ Okinawa Institute of Science and Technology Graduate University, 1919-1 Tancha, Onna-son, Okinawa 904-0495, Japan; ^2^ Faculty of Tropical AgriScience, Czech University of Life Sciences, Kamýcká 129, 165 00 Prague 6 Suchdol, Czech Republic; ^3^ Laboratory of Trophic and Isotopes Ecology (LETIS), UR FOCUS, 13 allee du six aout, University of Liège, 4000 Liege, Belgium; ^4^ Institute of Geochemistry, Mineralogy and Mineral Resources, Faculty of Science, Charles University, Albertov 6, 128 00 Prague, Czech Republic; ^5^ Evolutionary Biology and Ecology, Université Libre de Bruxelles, Avenue F.D. Roosevelt 50, CP 160/12, B-1050 Brussels, Belgium

**Keywords:** Isoptera, biogeography, ecology, feeding group, stable isotopes, mitogenomes

## Abstract

Termites feed on vegetal matter at various stages of decomposition. Lineages of wood- and soil-feeding termites are distributed across terrestrial ecosystems located between 45°N and 45°S of latitude, a distribution they acquired through many transoceanic dispersal events. While wood-feeding termites often live in the wood on which they feed and are efficient at dispersing across oceans by rafting, soil-feeders are believed to be poor dispersers. Therefore, their distribution across multiple continents requires an explanation. Here, we reconstructed the historical biogeography and the ancestral diet of termites using mitochondrial genomes and δ^13^C and δ^15^N stable isotope measurements obtained from 324 termite samples collected in five biogeographic realms. Our biogeographic models showed that wood-feeders are better at dispersing across oceans than soil-feeders, further corroborated by the presence of wood-feeders on remote islands devoid of soil-feeders. However, our ancestral range reconstructions identified 33 dispersal events among biogeographic realms, 18 of which were performed by soil-feeders. Therefore, despite their lower dispersal ability, soil-feeders performed several transoceanic dispersals that shaped the distribution of modern termites.

## 1. Introduction

Termites are a clade of eusocial cockroaches comprising about 3000 extant species [1]. Although modestly diverse compared to other insects, they make up a significant part of the animal biomass and have essential decomposer functions in tropical and subtropical ecosystems [2,3]. Termites have been heuristically categorized into four feeding groups based on taxonomy, anatomy and worker gut content [4]. The feeding group I includes the nine families composing the paraphyletic lower termites, all feeding on wood or grass. The other three feeding groups exclusively include species of Termitidae, also known as higher termites, a clade nested within the lower termites, which feeds on a diverse array of substrates: species of feeding group II feed on wood, grass, microepiphytes, leaf-litter or detritus; species of feeding group III feed on decayed wood or soil with high organic matter content (humus); and species of feeding group IV feed on soil with low organic matter content. This feeding group classification can be simplified using nitrogen and carbon stable isotope ratios (δ^15^N and δ^13^C), which reflect both the species trophic position along the wood–soil decomposition gradient and whether the consumed organic matter was produced by C_3_ or C_4_ plants, respectively [5–7]. Stable isotope ratios allow the distinction of two categories, the wood-feeders, including groups I and II, and the soil-feeders, comprising groups III and IV [8]. In addition, the Macrotermitinae are often separated into a feeding group on their own (feeding group IIf), for their digestion is largely aided by *Termitomyces*, a fungus they cultivate in their nest [9].

Termites are distributed between 45°N and 45°S, and their diversity is centred in the tropics [9,10]. Termites have acquired their modern distribution through tens of dispersal events among biogeographical realms [11–16]. While some recent dispersal events have been mediated by human activities [17], most predate human origin. One natural means of dispersion consists in crossing land bridges that once connected biogeographic realms. As modern termites appeared 140–150 million years ago (Ma) [11,18–20], land dispersal followed by vicariance induced by continental drift is, in theory, a potential explanation for the distribution of early-diverging termite lineages. However, most termite dispersals occurred following the global cooling initiated approximately 34 Ma, at the Eocene–Oligocene boundary [21], long after the separation of modern continents [12–14,16,21]. More recent land connections, such as the *Gomphotherium* land bridge that connected Africa and Asia approximately 20 Ma [22], were presumably used by termites to move between continents [12,23]. However, many dispersal events cannot be explained by past land connections and necessarily involve transoceanic journeys.

While termites are poor flyers unable to cross large water bodies actively [24,25], they often live in wood pieces that can float across oceans as rafts [26,27]. The ability of termites to disperse by rafting in wood pieces is evidenced by their presence on remote oceanic islands, such as the Easter [28] and Galapagos islands [29]. Their dispersal abilities are also illustrated by the fauna of Krakatau, a group of Indonesian islands that were entirely defaunated in 1883 by a volcanic eruption. Surveys of the termite fauna performed more than a century after the eruption revealed that the islands were recolonized by a dozen wood-feeding species, demonstrating the high dispersal abilities of this feeding group [30,31]. By contrast, soil-feeders and fungus-growers did not recolonize the Krakatau islands despite their abundance on the neighbouring islands of Java and Sumatra, neither did they colonize most oceanic islands (e.g. [32,33]). Termite species feeding on wood are therefore better dispersers than those feeding on soil.

The better dispersal capabilities of wood-feeding termites compared with their soil-feeding relatives suggest that past transoceanic journeys were mostly performed by wood-feeding termites, and rarely, if ever, by soil-feeders [34]. However, several lineages of soil-feeding termites are distributed across multiple continents [9,10]. Two non-mutually exclusive hypotheses could explain their distribution. The first hypothesis posits that soil-feeding termite lineages on each continent descend from wood-feeding ancestors that dispersed across oceans and subsequently acquired a diet based on soil [35]. The second hypothesis posits that soil-feeders have rarely dispersed to new continents but, the few times they did, successfully established and diversified. While soil-feeders are rarely found inside non-decayed wood pieces able to float, some species build arboreal nests or were found inside epiphytes [36–38], which, attached to floating trees, could serve as rafts. In this study, we used a phylogenetic comparative approach to test whether soil-feeding evolved many times independently in each biogeographic realm from dispersing wood-feeding ancestors or, alternatively, whether modern soil-feeders descended from soil-feeding ancestors that dispersed and colonized new realms. We built termite phylogenetic trees using mitochondrial genomes and estimated termite diet using δ^13^C and δ^15^N. Using these two datasets, we reconstructed the ancestral diet and historical biogeography of termites. We estimated the likelihood of wood-feeders shifting diet and giving rise to soil-feeding lineages and the likelihood of wood- and soil-feeders dispersing across oceans using biogeographic models.

## 2. Methods

### (a) Biological samples

We sampled 324 termite colonies representing over 280 species (i.e. around 10% of extant diversity) for stable isotope ratio measurements and mitochondrial genome sequencing (electronic supplementary material, Data S1: Sheet 3). Samples were collected across five biogeographical realms (modified from [39]): the Australian, Afrotropical, Neotropical (including Panamanian), Oceanian and Oriental realms. We collected two samples from each colony: (i) one in 80% ethanol, stored at room temperature and used for morphological identification and stable isotope analyses and (ii) one in RNA-later, stored at −80°C whenever possible, and used for molecular analyses.

### (b) Isotope measurements

Carbon and nitrogen stable isotope ratios were obtained via continuous flow elemental analysis isotope ratio mass spectrometry (CF-EA-IRMS). Isotope ratios were expressed as δ values (in ‰) relative to the international standard Vienna Pee Dee Belemnite (VPBD) for carbon (δ^13^C) and atmospheric nitrogen (for δ^15^N). Analyses were performed in two different laboratories. The first series of samples were analysed at the University of Liège (Belgium) on a VarioMicro cube EA (Elementar, Langensbold, Germany) coupled in a continuous flow to an IsoPrime100 IRMS (Isoprime, Cheadle Hulme, UK). Certified reference materials (CRM) from the International Atomic Energy Agency (IAEA; Vienna, Austria) were used for calibrations, namely IAEA-CH-6 (sucrose) and IAEA-N-2 (ammonium sulfate) for carbon and nitrogen, respectively. Glycine (Merck, Darmstadt, Germany; δ^13^C = –47.5 ± 0.3‰; δ^15^*N* = 2.3 ± 0.3‰) was interspersed among samples and used as a secondary analytical standard. One sample was randomly selected for replication and measured every 15 analyses. Standard deviation on this replicated sample was 0.2‰ for δ^13^C and 0.3‰ for δ^15^N. The second series of samples were analysed at Charles University (Czech Republic) on a Thermo Flash 2000 EA connected to a Thermo Delta V Advantage IRMS (Thermo Scientific, Germany) in a Continuous Flow IV system. CRMs were IAEA-CH-3 (cellulose), IAEA-CH-6 and IAEA-600 (caffeine) for carbon, and IAEA-N-1 (ammonium sulfate), IAEA-N-2 and IAEA-NO-3 (potassium nitrate) for nitrogen isotopic calibration run during the same sequence. Raw δ were normalized to the scale using a CRM-based multiple-point linear regression. Analytical precision was within ±0.3‰ for δ^13^C and ±0.4‰ for δ^15^N.

Stable isotope ratios were measured on samples from 324 colonies, mainly from degutted workers. For samples with an insufficient number of workers, we used other castes to obtain enough dry tissues for stable isotope analyses (electronic supplementary material, data S1, sheet 1). This approach is justified by the strong correlation between the stable isotope ratios of workers and soldiers originating from the same colony (*n* = 56 pairs; δ^13^C: *R^2^* = 0.99, *F*_1,54_ = 6997, *p* < 0.001; δ^15^N: *R^2^* = 0.94, *F*_1,54_ = 817.8, *p* < 0.001; electronic supplementary material, figure S1). For colonies measured more than once, stable isotope measurements were averaged for downstream analyses (electronic supplementary material, data S1, sheet 2).

In addition to reflecting animal diet, stable isotope ratios also vary with geographical locations and must be standardized across sites [7]. Because stable isotope ratios of wood-feeding termites are usually indistinguishable from that of their food source [5,40], we performed baseline normalization by subtracting the average isotope ratio of wood-feeding species (excluding microepiphyte-feeders) calculated for each site to the stable isotope ratio values of every sample collected in that given site (electronic supplementary material, table S1; data S1, sheet 2). Classification into feeding groups and food types (wood, microepiphytes, litter, grass, humus, nest, soil and fungus) was based on the synthesis of [9] (see electronic supplementary material, data S1, sheet 2). We recognized three feeding groups: wood-feeders, which feed on wood, microepiphytes, litter or grass; soil-feeders, which feed on humus, termite nest or soil; and fungus-growers, which all belong to the Macrotermitinae and cultivate the cellulolytic fungus *Termitomyces* inside their nest.

### (c) Phylogenetic analyses

We used mitochondrial genomes to reconstruct two phylogenetic trees encompassing 324 termite colonies for which we measured stable isotope ratios. Most mitochondrial genomes used in this study have been previously published [11–14,16,41–43]. We sequenced 19 additional mitochondrial genomes using two methods. Whole-genomic DNA extraction was performed with the DNeasy Blood & Tissue extraction kit (Qiagen). For the first method, DNA was extracted from degutted specimens, and mitogenomes were amplified with two long-range PCR reactions using the TaKaRa LA Taq polymerase and the primer sets described in [13]. For the second method, DNA was extracted from worker whole bodies, and no PCR amplification steps were performed. One library was prepared for each sample separately. Libraries were paired-end sequenced on the Illumina platform. Mitochondrial genomes were assembled using metaSPAdes v. 3.13 [44]. In total, we used the mitochondrial genomes of 316 termite colonies for which we measured stable isotope ratios and eight mitogenomes from a different colony than the one used for isotope measurements (electronic supplementary material, data S1, sheet 3). In addition, the mitochondrial genomes of two roaches and five species belonging to early-diverging termite lineages were used as outgroups (electronic supplementary material, table S2).

The 13 protein-coding genes, two rRNA genes and 22 tRNA genes of the mitochondrial genomes were annotated with MitoFinder v. 1.4 [45]. Each gene was aligned separately using MAFFT v. 7.305 [46]. For protein-coding genes, codons were translated into amino acid sequences with the transeq function of EMBOSS v. 6.6.0 [47], and protein sequences were aligned with MAFFT. Protein alignments were back-translated into codon alignments using PAL2NAL v. 14 [48]. Alignments were concatenated using FASconCAT-G_v. 1.04.pl [49]. The concatenated alignment was split into five partitions: one partition for combined rRNA genes, one partition for combined tRNA genes and one partition for each codon position of protein-coding genes. Phylogenetic analyses were run with and without third codon position sites. For all analyses, the monophyly of Macrotermitinae + Sphaerotermitinae and non-Macrotermitinae non-Sphaerotermitinae Termitidae was enforced to match the transcriptome-based phylogeny of Bucek *et al*. [20]. Phylogenies reconstructed with mitochondrial data provided an alternative topology for this branching [11,12], presumably because of frequent introgression and incomplete lineage sorting in early termitid lineages.

Time-calibrated phylogenetic trees were reconstructed using BEAST v. 2.6.2 [50]. A GTR + G model of nucleotide substitution was selected for all partitions, in accordance with the results of ModelFinder implemented in IQ-TREE v. 1.6.12 [51,52]. The trees were given a Yule speciation process as prior. An uncorrelated lognormal relaxed clock was used to model rate variation among branches [53]. We used 15 fossils as minimum age constraints (electronic supplementary material, table S3). Fossil calibrations were implemented as exponential priors on node time with a 97.5% soft maximum bound [54]. Minimum age constraints and soft maximum bounds were obtained from PaleoBioDB v. 1.2 (https://paleobiodb.org; last accessed 8 April 2021). Markov chain Monte Carlo analyses were run for a total of 500 and 750 million generations for analyses without and with third codon positions, respectively. Trees and parameters were sampled every 50 000 steps. We visually inspected the trace file with Tracer v. 1.7 [55] and accordingly used a burn-in of 10% and 20% for analyses without and with third codon positions, respectively. Maximum clade credibility trees with median heights were obtained using TreeAnnotator (electronic supplementary material, data S2 and S3).

### (d) Comparative phylogenetic analyses of termite diet

All analyses were carried out in R v. 4.0.2 [56]. Phylogenetic trees were visualized and annotated using the packages ‘treeio’ [57] and ‘ggtree’ [58]. Outgroups were pruned out of the final trees.

We characterized the stable isotope compositions of wood-feeders, soil-feeders and fungus-growers. For each feeding group, we used a δ^13^C and δ^15^N biplot to model the isotope space using Bayesian standard ellipses implemented in the ‘SIBER’ and ‘SIAR’ packages [59,60]. A total of 4000 ellipses were simulated from two chains, each with 2 × 10^4^ iterations, 1 × 10^3^ burn-in and thinned by 10. We tested for differences among the isotope spaces of the three feeding groups using a MANOVA performed on δ^13^C and δ^15^N values. We also performed two univariate Kruskal–Wallis rank-sum tests followed by pairwise Nemenyi's tests implemented in the ‘PMCMR’ package. Finally, we performed a linear discriminant analysis using the ‘lda’ function of the package ‘MASS’.

We tested for a correlation between termite phylogenetic trees and δ^13^C and δ^15^N values using the Moran's I and Pagel's *λ* tests implemented in the ‘phylosignal’ package [61]. The signal was located on the phylogenetic trees using local Moran's I indices calculated with the ‘lipaMoran’ function of ‘phylosignal’.

We reconstructed the ancestral diet of termites using stable isotope ratios. Ancestral reconstructions of stable isotope ratios were performed with the ‘fastAnc’ function of the ‘phytools’ package [62], which implements a maximum-likelihood (ML) model for continuous traits. We also explored the adaptive landscape dynamics of isotopic data in the Termitidae using likelihood and Bayesian model fitting. We fitted three likelihood models, the Brownian motion (BM), the Ornstein–Uhlenbeck process (OU) and the time-dependent delta (δ) model of Pagel, with the ‘fitContinuous’ function of the ‘geiger’ package [63]. As opposed to the stochasticity of standard BM models, an OU process is representative of the evolution of a trait towards adaptive optima, while a δ process models a non-constant rate of trait evolution. We used the Bayesian reversible-jump algorithm from the package ‘bayou’ [64] for OU model fitting using a chain of 2 000 000 generations with a 10% sampling frequency and a burn-in of 30%. Macroevolutionary shifts and adaptive optima were mapped on the phylogenetic tree using the ‘phenogram’ function from ‘phytools’, as well as the ‘plotSimmap.mcmc’ and ‘phenogram.density’ functions from ‘bayou’.

We identified the nodes corresponding to dispersal events and their inferred diet in the Termitidae. We used both the ML reconstructions on isotopic data, as well as reconstruction using wood-feeders, soil-feeders and fungus-growers as categorical variables with the ‘ace’ function of the ‘ape’ package [65]. The ‘ace’ function implements the ML method of [66] with equal rates of transition among states. We classified samples into soil-feeders and wood-feeders using Bayesian standard intervals estimated from isotopic data with SIBER. We also used the ML method of [66] to reconstruct ancestral geographic ranges and identify dispersal events among biogeographic realms.

We tested whether diet is linked to dispersal abilities among biogeographic realms in Termitidae using the BioGeoBEARS package [67,68]. We considered five biogeographical realms (Australian, Afrotropical, Neotropical, Oceanian and Oriental). The maximum number of areas was set to two as no extant species of Termitidae occurs in more than two biogeographical realms. We conducted time-stratified analyses allowing dispersal probabilities to vary with geological events (electronic supplementary material, table S4). The dispersal probability between the Afrotropical and Oriental realms was higher 20 to 15 Mya owing to the *Gomphotherium* land bridge [22], and dispersal to New Guinea (Oceanian realm) was possible after the emergence of the New Guinea orogen 12 Ma [69]. We used three popular classes of biogeographic models: the dispersal-extinction-cladogenesis model (DEC) [70]; the DIVALIKE model, a ML implementation of the dispersal-vicariance analysis (DIVA) [71] and the BAYAREALIKE model, a ML implementation of BayArea [72]. These models mostly differ in the scale at which the cladogenetic processes (sympatry and vicariance) occur, with the exception of BAYAREALIKE in which vicariance is disallowed [68]. All models were run with and without the parameter ‘+ *j*’ that allows for jump dispersal events [68]. To test for a correlation between diet and dispersal abilities, we compared trait-independent biogeographic models, in which dispersal abilities are independent of diet, to trait-dependent biogeographic models, in which diet influences dispersal probability [67]. Our analyses assumed that the sister group of Termitidae is Oriental and wood-feeding. Analyses performed with the sister group of Termitidae considered as Afrotropical and soil-feeding yielded similar results. For the trait-dependent models, we fixed the dispersal probability multipliers of wood-feeders (*m*_1_ = 0.5) and estimated the dispersal probability of soil-feeders (*m*_2_) and fungus-growers (*m*_3_) within a [0,1] interval. Trait-independent and trait-dependent biogeographic models were run for every six models, making a total of 12 models. We used sample-size-corrected Aikake information criterion (AICc) weights and likelihood ratio tests (LRT) to evaluate the 12 models [67].

## 3. Results

Normalized δ^13^C and δ^15^N values ranged from −4.2‰ to 13.2‰ and −8.3‰ to 15.6‰, respectively (electronic supplementary material, Data S1: Sheet 3). Termite species of distinct feeding groups harboured different stable isotope compositions (electronic supplementary material, figure S2; MANOVA: Wilks’ *λ* = 0.46, *F_*2,321*_* = 75.97, *p* < 0.001), both for δ^13^C and δ^15^N values (Kruskal–Wallis tests; δ^13^C: *H_1_* = 63, *p* < 0.001; δ^15^N: *H_1_* = 175.63, *p* < 0.001). Wood-feeders differed from soil-feeders in both δ^13^C and δ^15^N values and from fungus-growers in δ^13^C only, while soil-feeders differed from fungus-growers in δ^15^N only (*post hoc* Nemenyi's test; all *p* < 0.001). Overall, δ^15^N was more informative than δ^13^C (LDA; LD1, δ^15^N: 96.88%; LD2, δ^13^C: 84.87%).

**Figure 1.**
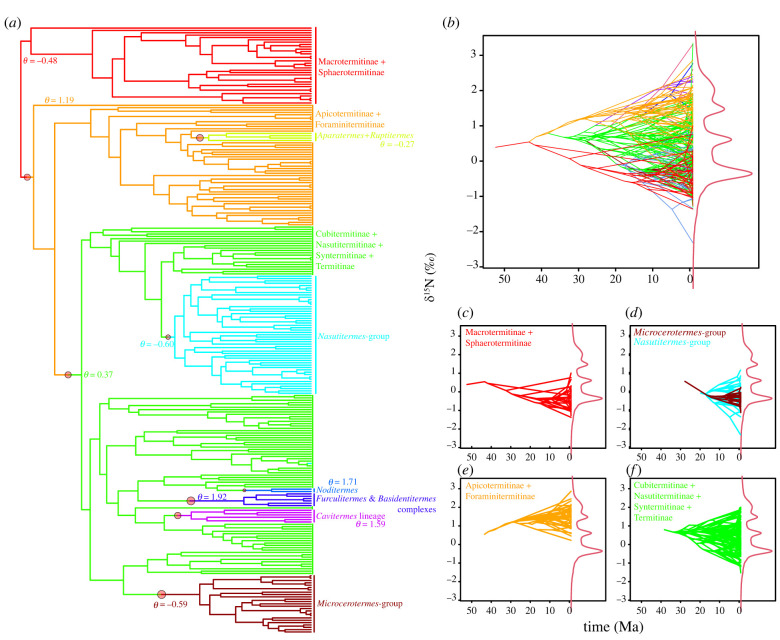
Macroevolutionary dietary shifts in the Termitidae were identified from centred and scaled δ^15^N values. (*a*) Location of the eight adaptive shifts (with posterior probability > 0.30) on the phylogeny of Termitidae (see electronic supplementary material, figure S7 for the location of all shifts). The shifts are indicated by circles, whose size reflects the posterior probability that a regime shift of *θ* occurred on that branch. Branch colours reflect convergent regimes in the adaptive optimum *θ*. (*b*–*f*) Phenograms of δ^15^N values with the posterior density of optima (curve in red) for (*b*) all lineages and for (*c*–*f*) the main dietary shifts. Shifts mostly reflect the interplay between the main termitid lineages with their diet.

The results of the analyses performed on both phylogenetic trees were congruent (electronic supplementary material, figures S3 and S4), indicating that our results are robust to phylogenetic inferences. For simplicity, we only present the results of the analyses performed on the phylogenetic tree reconstructed without third codon positions. The analyses performed on the phylogenetic tree reconstructed with third codon positions included are available in electronic supplementary material, figures S4, S6, S8 and table S6. Pagel's *λ* and Moran's I indices indicated strong phylogenetic signals both for δ^13^C (*λ* = 0.49, *p* = 0.001; *I* = 0.05, *p* = 0.004) and δ^15^N (*λ* = 0.85, *p* = 0.001; *I* = 0.12, *p* = 0.001). For δ^15^N, local significant signal was found for members of the Kalotermitidae (all wood-feeders exhibiting the lowest δ^15^N values), as well as for members of the Apicotermitinae and for the clade Cubitermitinae *+ Pericapritermes* group + *Termes* group (all soil-feeders exhibiting the highest δ^15^N values) (electronic supplementary material, figure S5). Our ML analyses indicated that the distribution of δ^15^N values fitted best an OU process, and Bayesian fitting in ‘bayou’ indicated eight significant macroevolutionary shifts (figure 1; electronic supplementary material, figure S7). Four of these eight shifts occurred during the emergence of major groups: non-macrotermitine non-sphaerotermitine termitids, the clade Cubitermitinae+Nasutitermitinae+Syntermitinae+Termitinae, the *Microcerotermes*-group and the *Nasutitermes*-group. Termite lineages were distributed along three main optima represented by (i) the comb-building Macrotermitinae+Sphaerotermitinae and the wood-feeding *Microcerotermes* and *Nasutitermes* (figure 1*c,d*); (ii) the ‘true’ soil-feeding Apicotermitinae and the Foraminitermitinae (figure 1*e*) and (iii) early-diverging Nasutitermitinae, the Syntermitinae, most Cubitermitinae and most Termitinae (figure 1*f*).

**Figure 2.**
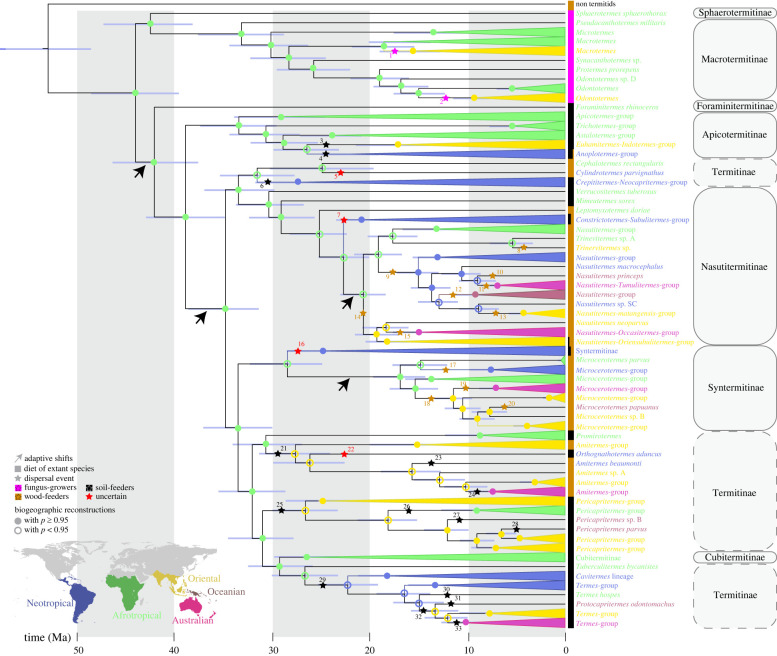
Biogeographic dispersals and diet of the Termitidae. Bayesian phylogenetic chronogram inferred from full mitochondrial genomes, without the third codon positions. Time scale is in millions of years, and node bars represent 95% credibility estimates of node time. Clades comprising species from a single biogeographic realm were collapsed (the full reconstruction is available in the electronic supplementary material, figure S3). Five biogeographic realms were recognized in this study (modified from [39]): circle colours indicate the biogeographic origin (full when the scaled likelihood of the most likely ancestral realm was above 95%, emptied otherwise), while text colours indicate the distribution of extant species. Dispersal events are indicated by stars, and their colours reflect the inferred diet of the dispersing termites (in brown, wood-feeders; in black, soil-feeders; in purple, fungus-growing termites which probably dispersed via a land bridge). Red stars indicate conflicts between the diet reconstruction methods (figure 3). The four major adaptive shifts identified by bayou (figure 1) are indicated with arrows. The diet of included extant species is indicated along the phylogeny (see electronic supplementary material, figure S3 for details).

**Figure 3.**
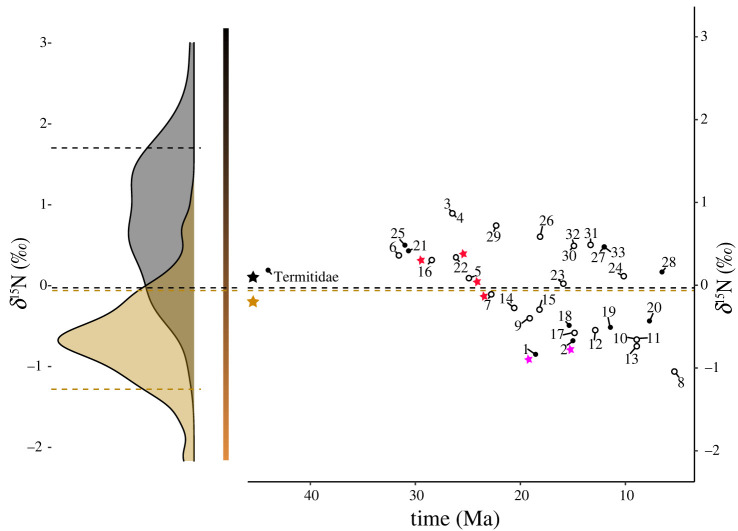
Ancestral diets were inferred at dispersal events between biogeographic realms. Smoothed density estimates of wood- and soil-feeders in extant termite species (left), and inferred diet in dispersing ancestors (right; *n* = 33 identified events). Ancestral reconstructions were performed from centred and scaled δ^15^N values (*n* = 33). Dashed lines represent the Bayesian standard intervals of wood-feeders (in brown; feeding on wood, microepiphytes, litter or grass) and soil-feeders (in black; humus, nest or soil) determined with SIBER and SIAR (purple stars indicate fungus-growing termites). Red stars indicate conflicts between reconstruction methods (continuous isotope data or discrete categories; conflict when the scaled likelihood for the most likely state was below 95% within the corresponding Bayesian standard intervals). Dispersal events are numbered, and empty circles indicate that the scaled likelihood of the most likely realm was below 95% (figure 2 for details).

Our ancestral range reconstructions inferred 33 dispersal events among the five studied biogeographic realms for Termitidae (figure 2). Of these, 15 dispersal events were performed by ancestors falling in the Bayesian standard intervals of extant wood-feeders (including fungus-growing Macrotermitinae), while 18 fell in the intervals of soil-feeders (figure 3). ML reconstruction from categorial classification indicated four ambiguous ancestral states over the 33 considered events. BioGeoBEARS analyses indicated that trait-dependent models better explained the historical biogeography of Termitidae than trait-independent models (table 1; electronic supplementary material, table S5). Trait-dependent models accrued 95.16% of AICc model weight. Cross-models AICc-weighted dispersal probabilities indicated that wood-feeders were the best dispersers (*m_1_* = 0.5), followed by the soil-feeders (*m_2_* = 0.2453) and the fungus-growers (*m_3_* = 0.1141). Finally, models that included founder jump dispersals (+ *j*) best explained the historical biogeography of Termitidae as these models accrued 100% of AICc model weight.

**Table 1.** Influence of trait state (1, wood-feeders; 2, soil-feeders; 3, fungus-growers) on dispersal rates using BioGeoBEARS from the BEAST2 analysis without third codon positions (see electronic supplementary material, table S5 for details), quantified as dispersal multipliers (*m*). Our analyses assumed that the sister group of all Termitidae are Oriental wood-feeders. For all analyses, *m*_1_ was fixed to 0.5. For trait-independent analyses, *m*_2_ and *m*_3_ were set to *m*_1_, while they were estimated within the [0,1] interval for trait-dependent ones. Likelihood ratio tests (LRT) for nested pairs of models are presented for the addition of the jump dispersal (*j*) and estimated trait state's dispersal multipliers. All 12 models were evaluated using corrected AICc weights. For LRT tests, significance parameter addition is indicated by asterisks (***, ** and *) at *p*-value cutoff of 0.001, 0.01 and 0.05, respectively (ns indicates non-significance at the 0.05 cutoff).

	base model	*m*_2_	*m*_3_	LRT (+ *j*)	LRT (+ *m*)	lnL	AICc weight (%)
Trait-independent	DEC + *t*_1–3_	*= m*_1_	*= m*_1_	NA	NA	−296.07	0
	DEC + *j* + *t*_1–3_			<0.0001***	NA	−248.38	1.24
	DIVALIKE + *t*_1–3_			NA	NA	−292.7	0
	DIVALIKE + *j* + *t*_1–3_			<0.0001***	NA	−247.35	3.5
	BAYAREALIKE + *t*_1–3_			NA	NA	−373.02	0
	BAYAREALIKE + *j* + *t*_1–3_			<0.0001***	NA	−250.97	0.09
Trait-dependent	DEC + *t*_1–3_ + *m*_1–3_	0.3064	0.3735	NA	0.0081**	−291.25	0
	DEC + *j* + *t*_1–3_ + *m*_1–3_	0.8701	0.2937	<0.0001***	0.2347	−246.94	0.6
	DIVALIKE + *t*_1–3_ + *m*_1–3_	0.2027	0.2033	NA	0.0081**	−287.88	0
	DIVALIKE + *j* + *t*_1–3_ + *m*_1–3_	0.2859	0.1111	<0.0001***	0.0044**	−241.93	90.35
	BAYAREALIKE + *t*_1–3_ + *m*_1–3_	0.1913	0.1665	NA	0.0847 ns	−370.55	0
	BAYAREALIKE + *j* + *t*_1–3_ + *m*_1–3_	0.3055	0.1001	<0.0001***	0.0025**	−244.99	4.21

## 4. Discussion

Soil-feeding termites have pantropical distribution, yet their ability to disperse across oceans is believed to be low [9,31,34,73]. We tested whether modern soil-feeding lineages descend from soil-feeding ancestors that carried out transoceanic dispersals or, alternatively, whether they descend from dispersing wood-feeding ancestors that secondarily evolved to feed on soil [12,35]. Our biogeographic models showed that dispersal abilities are linked to termite diet, with wood-feeders being better dispersers than soil-feeders and fungus-growers. However, despite their lower ability to disperse, we found that soil-feeders dispersed among biogeographic realms. Our ancestral diet reconstructions estimated that 18 of the 33 dispersal events were performed by soil-feeders, including some events that cannot be explained by land bridges, such as the colonization of the Neotropics by the African Apicotermitinae around 25 Ma (figure 2). Therefore, our results indicate that, despite their reduced ability to disperse, soil-feeders experienced long-distance over-water dispersal events that contributed to their modern distribution.

While our results provide strong evidence that both soil-feeders and fungus-growers dispersed across water gaps, they also indicate that wood-feeding termites are more efficient transoceanic dispersers. The ability of wood-feeders to disperse across large water gaps is probably linked to their ecology. Many species of wood-feeding termites nest inside wood pieces or harvest the lignocellulose of dead branches, sometimes retaining the ability to produce secondary reproductive when isolated from their parent colony [74]. Washed-up branches and trees, which are generated in large quantities during typhoons, tsunamis and other cataclysmic events, can serve as rafts carrying wood-feeding termites across oceans [26]. On the contrary, soil-feeding termites are rarely associated with wood items able to float.

About four-fifths of Termitidae are soil-feeders [1], a diet that is believed to have contributed to the ecological success of Termitidae. As previously suggested [20,75], our reconstructions of an ancestral diet indicated that soil-feeding was acquired early on in the evolution of Termitidae, before they spread out of Africa (figures 2 and 3). In support of the early origin of soil-feeding in Termitidae, Foraminitermitinae and the Apicotermitinae, two early-diverging lineages of Termitidae, are soil-feeding, and the first divergences within pantropical termitid lineages, such as the Nasutitermitinae and the Syntermitinae, are also soil-feeding (figures 2 and 3). The early origin of soil-feeding in Termitidae implies that soil-feeders had many opportunities to disperse across oceans.

While soil-feeding termites are able to disperse across large water gaps, they do so less efficiently than wood-feeders, probably because most soil-feeding termites live in the soil or in decaying wood [76], which may not readily serve as rafts. Some species, however, build arboreal nests solidly fixed on trees [2,13,77]. The dispersal by rafting of entire arboreal nests attached to floating trees has been hypothesized for wood-feeding nasutes [31,78]. The same mechanism could explain the dispersal events of some soil-feeding taxa building arboreal nests, such as some Apicotermitinae and Syntermitinae [2,13,37], which could survive long transoceanic journeys feeding on the organic matter stored in their nests. Soil-feeders have also been found occasionally in the suspended soil attached to palm tree crowns and canopy ferns and bromeliads [34], which could constitute another dispersal vehicle. In addition, some may have dispersed inside wood pieces akin to their wood-feeding relatives. Indeed, many species qualified as soil-feeders display intermediate stable isotope ratios and feed at the wood–soil interface on highly rotten wood that lost its wood structure [76]. While rotten wood may not float as a raft, rotten branches attached to floating trunks may transport soil-feeding termites displaying intermediate stable isotope ratios, such as species of the *Termes* and *Pericapritermes* groups [9] (electronic supplementary material, figure S5). The widespread pantropical distribution of these groups may therefore be explained by their feeding ecology, possibly enabling them to colonize new biogeographic regions more easily than ‘true’ soil-feeding taxa feeding on, and living in, the soil (figure 2). These considerations may also pertain to the Apicotermitinae, as *Adaiphrotermes*, an Afrotropical genus sister to the Oriental and Neotropical apicotermitines [42] not included in our analyses, is often found within sound wood [79]. In any case, some soil-feeding lineages appear to be weak colonizers and never dispersed outside Africa, such as the basal Apicotermitinae, *Promirotermes* and the Cubitermitinae. Similarly, the groups containing true soil-feeders that were established outside Africa, such as the Neotropical *Subulitermes* group, never dispersed outside the Neotropical realm.

Although we identified a number of dispersal events performed by soil-feeding termites, some soil-feeding lineages clearly descend from wood-feeding dispersing ancestors. This is the case for all dispersal events performed by nasutitermitines, with the probable exception of the Neotropical *Subulitermes*-group. One example is the clade including the soil-feeding *Oriensubulitermes* (figure 2), which is endemic to the Oriental realm and descends from a wood-feeding ancestor. Another example is *Malagasitermes*, a soil-feeding nasute endemic to Madagascar and nested within wood-feeding nasutes [15,35], which was not included in our analyses. Nevertheless, our results indicate that soil-feeders performed transoceanic dispersals, contributing to their modern pantropical distribution.

While incomplete, our sampling is comprehensive, with all termitid lineages sampled at a similar depth. Hence, our sampling is representative of the extant termite diversity [1] and the overall dispersal patterns of termites. Increasing taxon sampling could reveal additional dispersal events among both wood- and soil-feeders, or improve our inferences of the diet of the dispersing ancestors. For example, the inclusion of *Adaiphrotermes* may show that Oriental and Neotropical apicotermitines descend from wood-feeding dispersing ancestors. Our ancestral state reconstructions portrayed a high variability of δ^15^N values in modern species that gradually averaged in the past (figure 1*b*). These results must be taken with a grain of salt as ancestral reconstruction methods on continuous traits have been shown to sometimes produce inaccurate predictions, unsupported by fossil evidence [80–82]. In theory, isotopic measurements can be performed on chitinous fossils allowing inference of paleodiets [83]. The feeding habits of fossil termites could also be inferred by observing worker-imago mandibles, which are generally of grinding type in wood-feeders and pounding type in soil-feeders [84]. Analyses of fossil taxa are needed to confirm whether termite diet diversity has gradually increased since Termitidae first appeared.

## Data Availability

Electronic supplementary material is available on FigShare [85], and on the Dryad Digital Repository: https://doi.org/10.5061/dryad.41ns1rngs [86]. The mitochondrial genomes generated in this study are available on GenBank under accession nos. OK163842–OK163858 and OL469804–OL469805 (electronic supplementary material, data S1, sheet 3).
